# PFOS induces proliferation, cell-cycle progression, and malignant phenotype in human breast epithelial cells

**DOI:** 10.1007/s00204-017-2077-8

**Published:** 2017-10-23

**Authors:** Paula Pierozan, Oskar Karlsson

**Affiliations:** Department of Pharmaceutical Biosciences, Uppsala University, Uppsala, Sweden

**Keywords:** Perfluorooctanesulfonic acid, MCF-10A cells, Breast cancer, Cell transformation, CDK4, p53

## Abstract

Perfluorooctanesulfonic acid (PFOS) is a synthetic fluorosurfactant widely used in the industry and a prominent environmental toxicant. PFOS is persistent, bioaccumulative, and toxic to mammalian species. Growing evidence suggests that PFOS has the potential to interfere with estrogen homeostasis, posing a risk of endocrine-disrupting effects. Recently, concerns about a potential link between PFOS and breast cancer have been raised, but the mechanisms underlying its actions as a potential carcinogen are unknown. By utilizing cell proliferation assays, flow cytometry, immunocytochemistry, and cell migration/invasion assays, we examined the potentially tumorigenic activity of PFOS (100 nM–1 mM) in MCF-10A breast cell line. The results showed that the growth of MCF-10A cells exposed to 1 and 10 µM PFOS was higher compared to that of the control. Mechanistic studies using 10 µM PFOS demonstrated that the compound promotes MCF-10A proliferation through accelerating G_0_/G_1-_to-S phase transition of the cell cycle after 24, 48, and 72 h of treatment. In addition, PFOS exposure increased CDK4 and decreased p27, p21, and p53 levels in the cells. Importantly, treatment with 10 µM PFOS for 72 h also stimulated MCF-10A cell migration and invasion, illustrating its capability to induce neoplastic transformation of human breast epithelial cells. Our experimental results suggest that exposure to low levels of PFOS might be a potential risk factor in human breast cancer initiation and development.

## Introduction

Perfluorooctanesulfonic acid (PFOS), is a man-made fluorosurfactant that has been used as lubricant, component of polishes, paints, paper, textile coatings, food packaging, and fire-retardant foams (Lindstrom et al. 2011). PFOS and PFOS-containing products are released to the environment, contaminating soil, sediment, sludge, and water (Boulanger et al. 2005; Skutlarek et al. 2006). Due to its water solubility and resistance to biological degradation, PFOS is mobilized and released from landfills in water bodies, and bio-accumulates in food chains (Giesy et al. 2001; Parsons et al. 2008). Humans are exposed to PFOS via oral, inhalation, and dermal routes. Studies report that, for the general population, ingestion of fish and drinking water are the main sources of PFOS exposure. Bioaccumulation occurs in humans, leading to a half-life of 5.4 years in serum (Olsen et al. 2007). PFOS are detected in concentrations of up to 48 ng/ml in human samples such as serum and breast milk, depending on the country and dietary intake (Karrman et al. 2007; Sundstrom et al. 2011). PFOS exposure can result in a variety of toxic effects, including liver toxicity, developmental toxicity, and immunotoxicity (Chang et al. 2008; Saikat et al. 2013). Adverse effects on reproductive organs have also been reported (Saikat et al. 2013). In addition, PFOS is suspected to be an endocrine disruptor with estrogenic activity (Jensen and Leffers 2008), and may contribute to the risk of breast cancer (Bonefeld-Jorgensen et al. 2011), but the potential for PFOS to contribute to endocrine disruption in mammalians is not well defined.

Hormonally active chemicals, referred to as endocrine disruptors, may be implicated in breast cancer etiology through promotional mechanisms, as well as by affecting mammary gland development (Kortenkamp 2006). Among them, both estrogens and non-estrogenic endocrine disruptors are considered to play critical roles in human breast carcinogenesis (Brody et al. 2007). As a result, investigation of hormonally active compounds in commercial products and pollution is a priority.

Several studies have reported that exposure to PFOS may induce cancer development (Gallo et al. 2012; Olsen et al. 1999, 2007). For example, a case–control study in the Greenland Inuit population revealed an increased breast cancer risk in relation to the level of serum PFOS (Bonefeld-Jorgensen et al. 2011). Moreover, PFOS is suspected to have estrogenic activity and in vitro studies showed that PFOS might have the ability to interact with estrogen receptors (ER)-α and ERβ and enhance ER-dependent transcriptional activation (Benninghoff et al. 2011; Du et al. 2013; Kjeldsen and Bonefeld-Jorgensen 2013). However, others have reported contradictory data on its estrogenicity and the potential for PFOS to contribute to breast cancer is not well studied (Ishibashi et al. 2007; Maras et al. 2006).

The possible mechanisms by which PFOS may affect the breast tissue can be assessed by animal and cell models. MCF-10A is a subline of the spontaneously immortalized human breast epithelial line that is derived from human fibrocystic mammary tissue, with characteristics of normal breast epithelium (Soule et al. 1990). These characteristics make the MCF-10A a valuable in vitro model for studying normal breast cell function and the potential of external factors to induce tumor transformation.

In the present study, we investigated the effects of PFOS exposure in the human normal breast epithelial cell line, MCF-10A, at various concentrations (100 nM–1 mM) and different time points (24, 48 and 72 h). Cell viability, cell counting, and cell-cycle analysis were applied to investigate the effects of PFOS on cell proliferation. The differential expression of important cell-cycle regulatory proteins was also analyzed and a transwell assay was used to evaluate PFOS-induced cell migration and cell invasion capability. Finally, the effect of PFOS on ER activation and ERα and ERβ protein expression was evaluated. This study will help to provide insight into the mechanisms involved in human toxicity of PFOS in breast cells and its involvement in breast cancer.

## Materials and methods

### Chemicals

Dimethylsufoxide (DMSO), pharaformaldehyde, 4′,6-diamidino-2-phenylindole dihydrochloride (DAPI), Triton X-100, Propidium iodide (PI), DNAse-free RNAse A, Perfluorooctanesulfonic acid potassium salt (PFOS), cholera toxin (CT), insulin, epidermal growth factor (EGF), and hydrocortisone were obtained from Sigma-Aldrich (St Louis, MO, USA). Horse serum, penicillin–streptomycin (P/S), Dulbecco’s Phosphate-Buffered Saline (PBS), Dulbecco’s Modified Eagle’s Medium (DMEM), and trypsin solution (0.05%) were obtained from Gibco (Invitrogen, Paisley, UK). p53 monoclonal (DO-7), CDK6 monoclonal (75B9), CDK4 monoclonal (DCS-31), and p21 monoclonal (R.229.6) antibodies were obtained from ThermoFisher Scientific (Rockford, IL, USA). P27 Kip1 (D69C12) and Cyclin D1 (92G2) antibodies were obtained from Cell Signaling (Danvers, MA, USA). The secondary antibodies alexa-fluor 555 goat anti-mouse IgG or 488 goat anti-rabbit IgG, and the blocking agent (normal goat serum) were obtained from Molecular Probes, Invitrogen. Matrigel Basement Membrane Matrix was obtained from Corning (New York, NY, USA). ERα (sc-8002) and ERβ (sc-8974) monoclonal antibodies were obtained from Santa Cruz Biotechnology (Bergheimer, HD, DE). ICI 182,780 was obtained from Tocris Bioscience (Avonmouth, Bristol, UK).

### Cell culture

MCF-10A cells were obtained as frozen vials from the American Type Culture Collection (ATCC, Manassas, VA, USA). Cells were maintained as a monolayer in 10 cm^2^ tissue-culture plastic flasks containing 10 ml of growth medium, trypsinized (0.05%) and split 1:5 every 3 days. Complete growth medium consisted of Dulbecco’s Modifies Eagle Medium with F-12 (DMEM/F-12; GIBCO, Invitrogen, Paisley, UK) supplemented with horse serum (5%), EGF (20 ng/ml), hydrocortisone (0.5 mg/ml), CT (100 ng/ml), insulin (10 mg/ml), and 5 ml P/S. Cell cultures were maintained at 37 °C and 5% CO_2_ in a humidified incubator.

### Exposure of MCF-10A cells to PFOS

MCF-10A cells were trypsinized and resuspended in growth medium, plated in 96-well tissue-culture plates (2 × 10^4^ cells/well), and allowed to attach for 24 h in a 5% CO_2_ humidified incubator at 37 °C. After 24 h, the cells were treated with different PFOS concentrations (0–1 mM) dissolved in DMSO and assay medium (growth medium without horse serum and EGF). Controls were exposed to 0.1% DMSO only. The cells were incubated for 24, 48, and 72 h. The experiments were repeated three times.

### 3-(4,5-dimethyl-2-yl)2,5-diphenyl-2H-tetrazolium bromide (MTT) assay

MCF-10A cells were treated with different concentrations of PFOS (0–1 mM) for 24, 48, and 72 h, with eight wells for each treatment and three independent experiments. Cell viability was measured by the MTT assay, which is based on the conversion of the tetrazolium salt to the colored product formazan. Only viable cells are able to reduce MTT. In brief, 0.5 mg MTT was added to each well of the 96-well plates (containing 100 μl medium and cells) 1 h before the end of incubation with PFOS. The supernatant was then separated, and 100 μl dimethylsulfoxide (DMSO) was added to each well, followed by incubation and shaking for 10 min. The formazan product generated during the incubation was solubilized in DMSO and measured at 490 and 630 nm using a Polarstar Optima microplate reader (Bmg Labtech, Offenburg, Germany).

To investigate the participation of estrogen receptors on the PFOS effects, the cells were preincubated 30 min with the ER receptor blocker ICI 182,780 (100 nM), followed by incubation with PFOS 10 µM for 72 h, before the MTT assay was performed.

### Cell counting by DAPI staining

MCF-10A cells were treated with different concentrations of PFOS (0–1 mM) for 24, 48, and 72 h, with three replicates of each treatment. After the treatment, cells were fixed with 4% paraformaldehyde for 30 min and permeabilized with 0.1% Triton X-100 in PBS for 5 min at room temperature. Cells were stained with DAPI (0.25 mg/ml) for 10 min at room temperature followed by two washes with PBS. Cells were viewed in an ImageXpress Micro XLS Widefield High-Content Analysis System (Molecular Devices, Sunnyvale CA, USA), and images analyzed with the SoftMax Pro Software after digital acquisition (Molecular Devices, Sunnyvale CA, USA).

### Analysis of cell-cycle phases and proteins involved in cell-cycle regulation

Cells were processed for PI staining and flow cytometry as described previously (Pozarowski and Darzynkiewicz 2004). After 24, 48, and 72 h, the supernatant was collected and monolayer exposed to 0.05% trypsin/EDTA. Cells were centrifugated at 92 g for 5 min, and resuspended in 1 ml PBS. After this, pelleted cells were fixed by dropwise adding 2 ml 70% ice-cold ethanol while vortexing and then kept on ice for 1 h before storage at 4 °C. The samples were stored for at least 48 h before analysis to allow leakage of fragmented DNA from apoptotic cells and their identification as a fraction with DNA content less than G_0_/G_1_, referred to as the sub-G_0_/G_1_ fraction. On the day of analysis, fixed cells were kept on ice and washed twice in PBS, and each sample incubated with 1 ml PI (50 mg/ml) and RNAse A (50 ng/ml) in PBS for 3 h at 4 °C in the dark. Forward and light scatter data were collected in a linear mode. Fluorescence data for 10,000 cells per sample were collected in the FL3 channel on a linear scale. Side- and forward-light scatter parameters were used to identify the cell events and doublets were excluded using gating. Samples were analyzed using a Cytoflex flow cytometer (Beckman Coulter Ltd., Brea, CA, USA). Cells in different cell-cycle phases were presented as a percentage of the total number of cells counted.

To evaluate the effects on proteins involved in cell-cycle regulation, cells were incubated with anti-Cyclin D, anti-CDK4/6, and anti-p21/27/53 antibodies, and analyzed by flow cytometry. Cells were fixed with 0.4% paraformaldehyde and 1.5 × 10^6^ cells were incubated overnight with different antibodies diluted in a block solution (BSA 1%/Triton X-100 2%) in a saturated concentration, followed by incubation with goat anti-rabbit IgG 488 or goat anti-mouse IgG 555 as secondary antibody for 1 h. Flow cytometry was performed using a Cytoflex flow cytometer (Beckman Coulter Ltd., Brea, CA, USA). Side and forward scatter of aggregates or lysed cells were determined using log scale SSC/FSC plots with thresholds. Voltage settings for the SSC, FSC, and the fluorescent filters were kept constant for all experiments described. The results are expressed as mean fluorescence intensity compared with the controls (% of controls) for 10,000 cells per sample.

### Immunocytochemistry

MCF-10A cells were treated with 10 µM PFOS 72 h and immunocytochemistry was performed as previously described (Pierozan et al. 2016). Briefly, cells plated on glass coverslips were fixed with 4% paraformaldehyde for 30 min and permeabilized with 0.1% Triton X-100 in PBS for 5 min at room temperature. After blocking with BSA 5%, cells were incubated overnight with primary antibodies (1:1000). PBS washes and incubation with specific secondary antibodies conjugated with alexa-fluor 488 or 555 were performed for 1 h. Negative control reactions were performed by omitting the primary antibody with no observed fluorescence. Nuclei were stained with DAPI (0.25 mg/ml). Cells were examined in an Olympus inverted microscope (Olympus, Tokyo, Japan), and images were collected with a 20× objective using constant intensity settings and exposure time for all samples. The intensity of the cell fluorescence was measured using the Image J Software (ImageJ2), and the fluorescence intensity was estimated as the difference between the measured fluorescence of the cells and the background.

### Migration and invasion assay

Transwell migration and invasion assays were conducted in 96-well plates with membrane inserts (8 µm pore-size) (Corning, New York, NY, USA). Cells were treated with 10 µM PFOS for 72 h. After that, 5 × 10^5^ cells were resuspended in 50 µl of assay medium and seeded in the upper chamber of transwells with (invasion assay) or without (migration assay) Matrigel Matrix (200 µg/ml). The lower chamber contained 100 µl growth medium. Cells were incubated for 24 h at 37 °C in a humidified atmosphere with 5% CO_2_. At the end of incubation, non-invasive cells in the upper chamber were removed and invasive cells in the bottom were fixed with 4% formaldehyde and stained with DAPI and then were counted as described in the Sect. “Cell counting by DAPI staining” of “Materials and methods”. Cells were viewed in an ImageXpress Micro XLS Widefield High-Content Analysis System (Molecular Devices, Sunnyvale, CA, USA), and images were analyzed with the SoftMax Pro Software after digital acquisition (Molecular Devices, Sunnyvale, CA, USA).

### Western blot analysis

For the evaluation of ER protein levels, cells were exposed to 10 µM PFOS or 10 nM 17β-estradiol (E2-positive control) for 72 h. Cells were then washed with ice-cold PBS and lysed with Laemmli lysis buffer. The protein concentration in cell lysates was determined using bicinchoninic acid protein assay (Smith et al. 1985). Equal amounts of protein (30 µg) were separated by sodium dodecyl sulfate–polyacrylamide gel electrophoresis (SDS-PAGE) on 10% gel and transferred (Mini Trans-Blot Electrophoretic Transfer Cell-Bio-Rad, Hercules, CA, USA) to polyvinylidene difluoride membranes for 1 h at 100 V in transfer buffer (25 mM Triz, 192 M glycine, 20% methanol and 0.1% SDS). The blot was then washed for 20 min in Tris-buffered saline (TBS; 500 mM NaCl, 20 mM Trizma, pH 7.5), followed by 2 h incubation in blocking solution (TBS plus 5% defatted dry milk). After the incubation was finished, the blot was washed twice for 5 min with blocking solution plus 0.05% Tween-20 (T-TBS) and incubated overnight at 4 °C in blocking solution containing monoclonal antibodies diluted 1:1000. The blot was then washed twice for 5 min with T-TBS and incubated for 2 h in a solution containing peroxidase-conjugated rabbit anti-mouse IgG-diluted 1:2000 or peroxidase-conjugated anti-rabbit IgG-diluted 1:2000. The blot was washed twice with T-TBS for 5 min and twice with TBS for 5 min. The blot was developed with the chemiluminescence ECL kit (Little Chalfont, UK).

### Statistical analysis

The results were presented as mean ± standard deviation (SD) for each experimental group of at least three individual samples. Differences between the control were analyzed by one-way analysis of variance (ANOVA) followed by Tukey–Kramer multiple tests using Graphpad Prism 7 software.

## Results

### PFOS-induced cell death and proliferation are dependent on the time and concentration

**Fig. 1:**
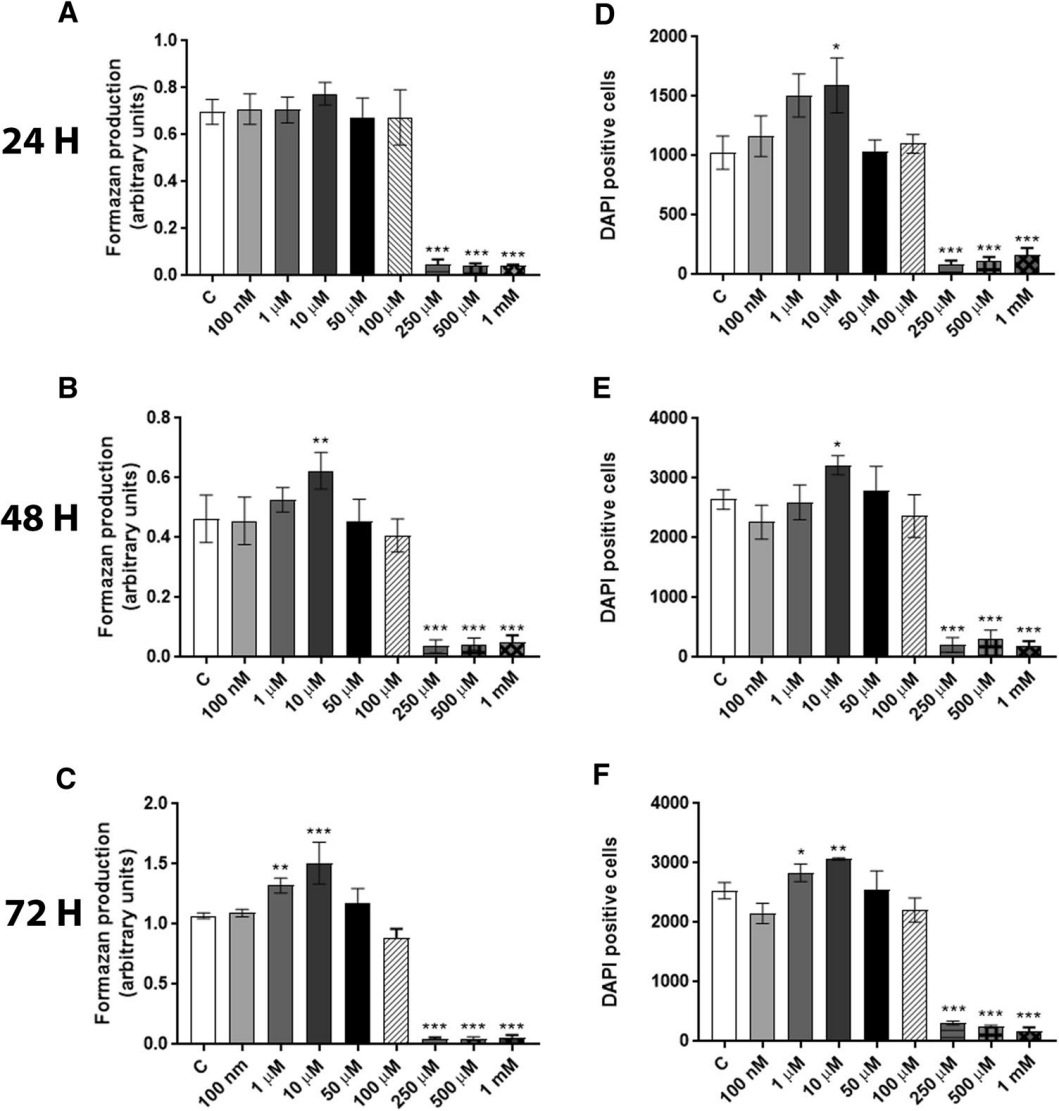
Effects of PFOS on the viability of MCF-10A cells. The cells were exposed to 0–1 mM PFOS for 24, 48, and 72 h. The controls were incubated with 0.1% dimethylsulfoxide (DMSO). The viability was determined by MTT assay (**a**, **b,** and **c**) and DAPI staining (**d**, **e**, and **f**). Values represent mean ± SD from three independent experiments. Statistically significant differences from control are indicated as follows ***p < 0.001; **p < 0.01 and *p < 0.05 (One-way ANOVA followed by the Tukey–Kramer test)

To determine whether PFOS exposure can induce cell death or proliferation, cells were incubated with 0–1 mM PFOS for 24, 48, and 72 h, and cell viability determined by the MTT assay. We found significantly decreased cell viability in PFOS concentrations equal to 250 µM or higher at all time points (Fig. 1a, c). In contrast, exposure to 10 µM PFOS for 48 h (Fig. 1b) and 1 and 10 µM PFOS for 72 h (Fig. 1c) increased MTT production, suggesting an increase in cell proliferation. To confirm this observation, the number of cells was determined using DAPI staining and fluorescence microscopy. The results showed that PFOS caused an increase in the number of cells at 10 µM 24 h and 48 h exposure (Fig. 1d, e) as well as 1 and 10 µM at 72 h (Fig. 1f).

### PFOS affects the cell cycle in MCF-10A cells

To determine the effects of PFOS exposure on cell-cycle distribution, cells were stained with PI and analyzed by flow cytometry. Table 1 shows the mean percentage of gated cells for each DNA content fraction (G_0_/G_1_, S, G_2_/M) following 24, 48, and 72 h exposure to 10 µM PFOS. PFOS exposure reduced the percentage of cells in G_0_/G_1_ phase and increased the percentage of cells in S phase at all time points.

**Table 1:** Effects of PFOS (10 µM) on MCF-10A cell cycle

	24 h			48 h			72 h		
	G0/G1	S	G2/M	G0/G1	S	G2/M	G0/G1	S	G2/M
Control	68.3 ± 15	17.0 ± 10	10 ± 6.17	51.3 ± 6.7	34.0 ± 6.8	14.7 ± 0.3	56.0 ± 5.4	33.1 ± 7.9	10.9 ± 2.6
PFOS	54.4 ± 7.8***	36.2 ± 3.6**	13.4 ± 2.5	36.2 ± 1.3**	57.2 ± 2.3***	6.6 ± 1.2***	32.9 ± 1.9***	57.7 ± 7.5**	9.4 ± 6.0

Results as percentage of total events (10 000 events)

Statistically significant differences from control are indicated as follows ***p < 0.001 and **p < 0.01 (Student’s *t* test)

### PFOS alters the levels of proteins involved in cell-cycle regulation

**Fig. 2:**
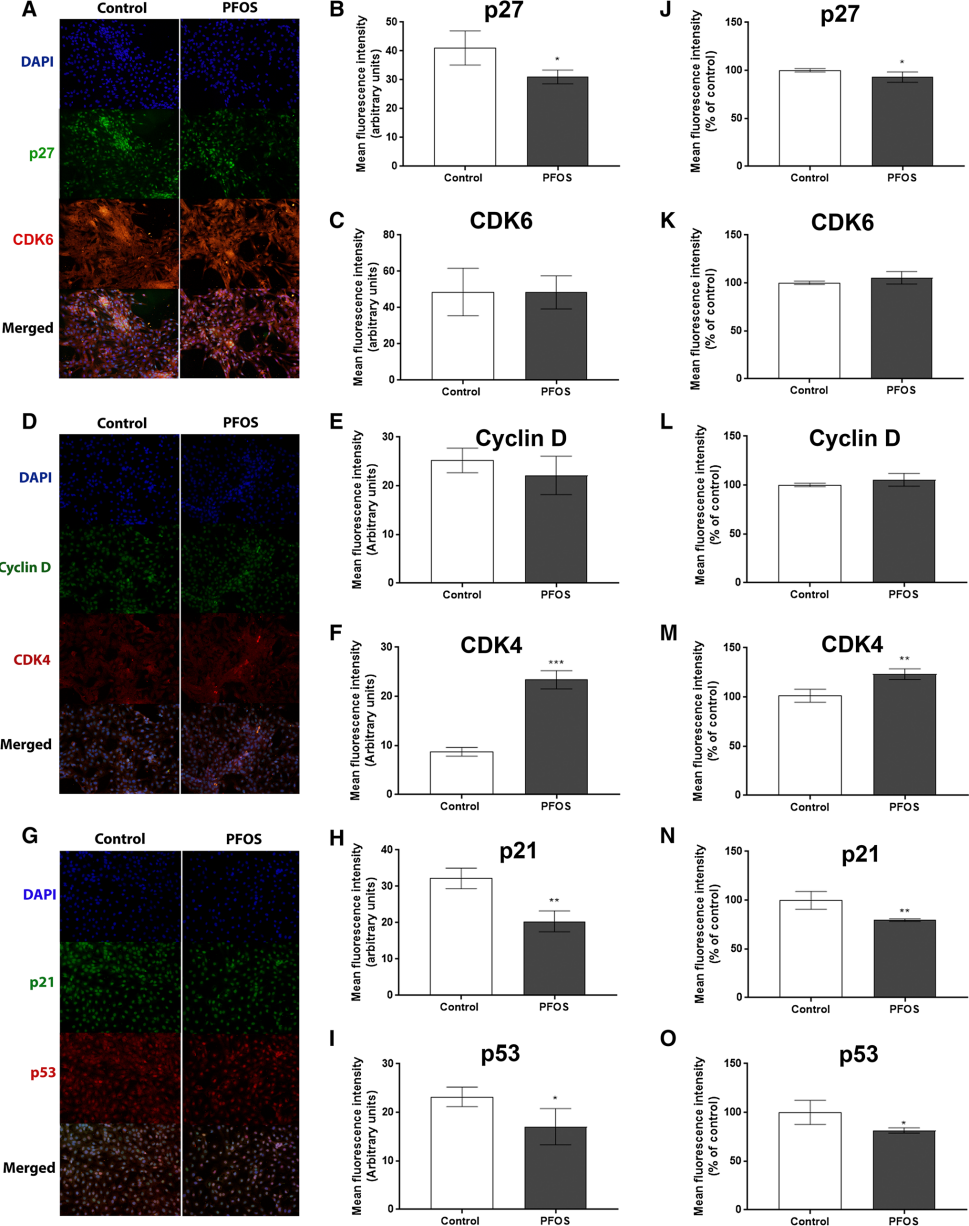
Effects of PFOS on the levels of proteins involved in cell-cycle regulation. The cells were exposed to 10 µM PFOS for 72 h before immunocytochemistry and flow cytometry was performed. Representative images of PFOS-treated cells immunostained with p27 and CDK6 (**a**), Cyclin D1 and CDK4 (**b**), and p21 and p53 (**c**). Mean fluorescence intensity was analyzed from immunocytochemistry (**b**–**i**) and flow cytometry (**j**–**o**) as described in Materials and methods section. Values represent mean ± SD from three independent experiments. Statistically significant differences from control are indicated as follows: ***p < 0.001; **p < 0.01 and *p < 0.05 (Student’s *t* test)

To investigate mechanisms involved in PFOS-induced cell proliferation in MCF-10A cells, the levels of the cyclin-dependent kinases (CDKs) CDK4, CDK6, Cyclin D1, and their respective inhibitors (p27, p21, and p53) were analyzed by immunocytochemistry and flow cytometry and compared with control cells. The fluorescence microscopy images revealed a reduced p27, p21, and p53-fluorescence (Fig. 2a, b, g, h, and i), and an increased CDK4 fluorescence (Fig. 2d, f) in cells treated with PFOS, with no alteration in CDK6 and Cyclin D1-staining (Fig. 2a, c, d and e). The flow cytometry results confirmed the immunocytochemistry findings and showed a decrease in the mean fluorescence intensity in p27, p21, and p53-staining (Fig. 2j, n and o), and an increase in the mean fluorescence intensity in CKD4-staining (Fig. 2m) in PFOS-treated cells compared to the controls.

### PFOS promotes migration and invasion of MCF-10A cells

**Fig. 3:**
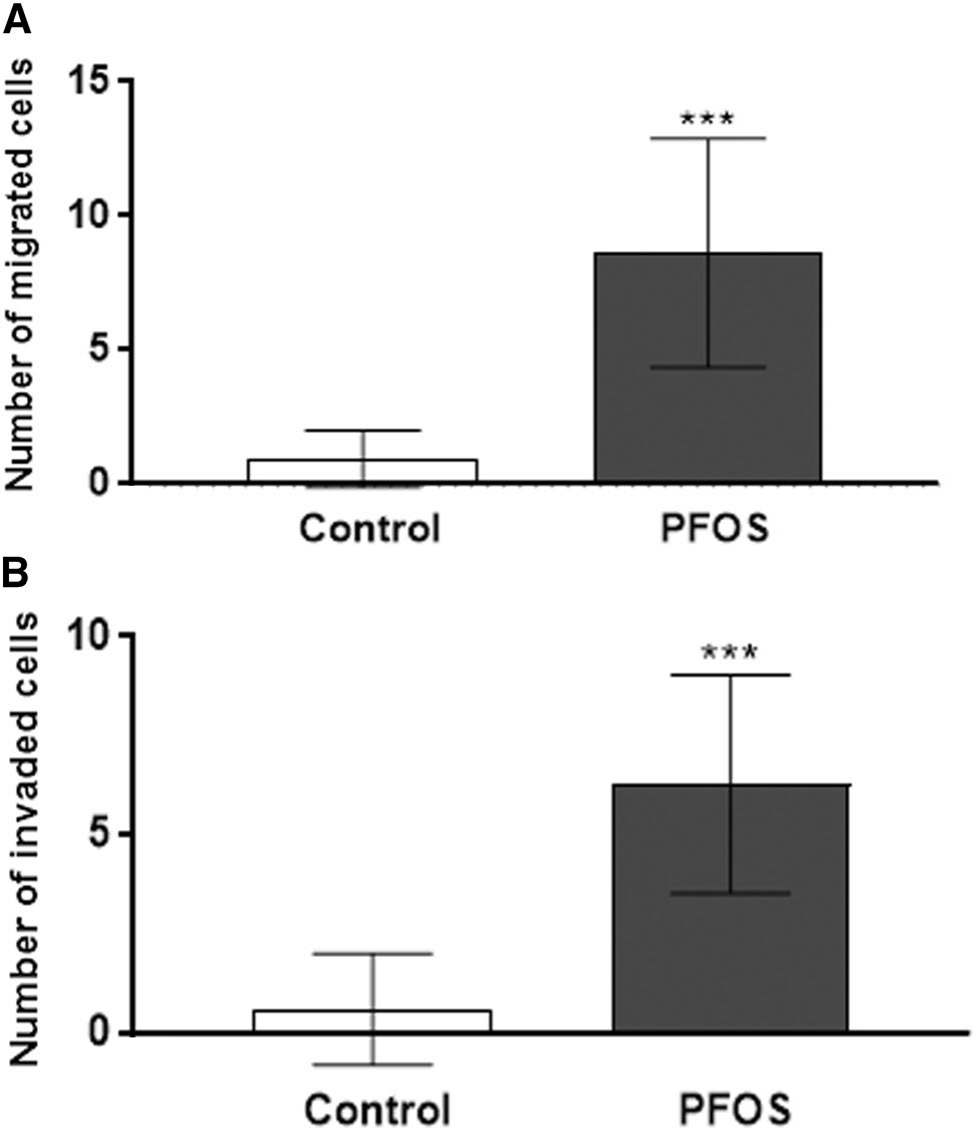
Effects of PFOS on MCF-10A cell migration and invasion capacity. Effects of PFOS on MCF-10A cell migration (**a**) and cell invasion (**b**) by a transwell assay. Migrated or invaded cells in the bottom were fixed with 4% formaldehyde and stained with DAPI and counted as described in the Materials and methods section. Values represent mean ± SD. Statistically significant differences from control are indicated as follows ***p < 0.001 (Student’s *t* test)

**Fig. 4:**
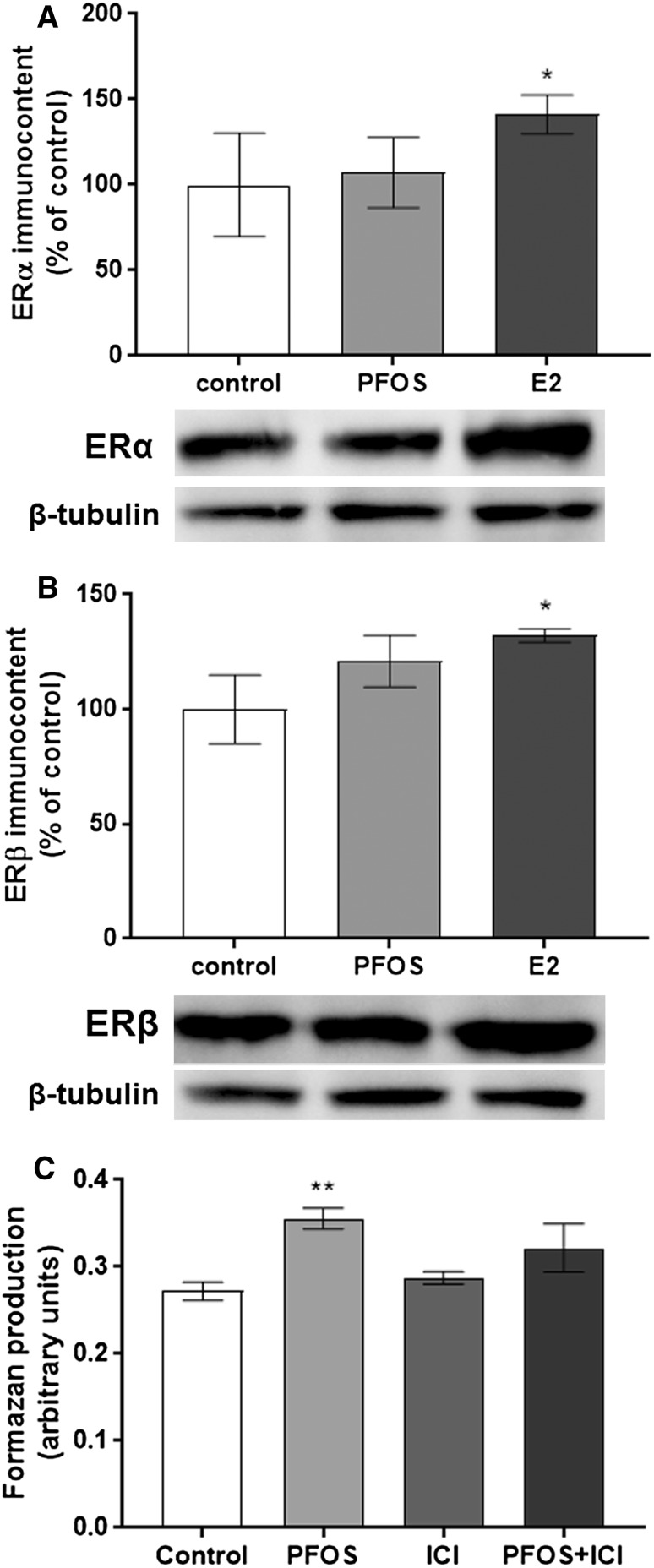
Involvement of the ER in the effects triggered by PFOS. Effect of PFOS and 17β-estradiol (E2-positive control) on ERα (**a**) and ERβ (**b**) protein levels in MCF-10A breast cells. The cells were exposed to 10 µM PFOS or 10 nM E2 for 72 h. β-tubulin was used as a loading control. Representative blots of three experiments are shown. The results of densitometry analysis are expressed as ER protein band density normalized to the density of β-tubulin bands. To determine the role of ER activation, cells were incubated with 100 nM ICI 182,780 followed by 10 µM PFOS, and the viability was determined by MTT assay (**c**). Data are reported as mean ± SD of three independent experiments. Statistically significant differences from control are indicated as follows **p < 0.01 and *p < 0.05 (One-way ANOVA followed by the Tukey–Kramer test)

To further investigate the effect of PFOS on cell aggression, we analyzed the effect of the compound on migration and invasion of MCF-10A cells using transwell migration and Matrigel invasion assays. As demonstrated in Fig. 4, the migration (Fig. 3a) and invasion capacity (Fig. 3b) of the MCF-10A cells were enhanced after treatment with PFOS, indicating that PFOS induces invasive abilities compared with the untreated control cells.

### Effect of PFOS on ERα and ERβ protein levels and ER activation in MCF-10A cells

Since it has been shown that PFOS can interact directly or indirectly with estrogenic pathways (Kortenkamp 2006; Sonthithai et al. 2016), and MCF-10A cells can be transformed into a malignant phenotype by estrogen compounds (Hemachandra et al. 2012), we investigate the effects of PFOS on ER protein levels and the role of ER activation. In MCF-10A cells, 17β-estradiol (E2), used as positive control, increased ERα (Fig. 4a) and ERβ (Fig. 4b) levels after 72 h of exposure, while PFOS had no effect on ERα and ERβ levels. Moreover, treatment of MCF-10A cells with the ER receptor blocker, ICI 182,780 prevented only partially the stimulatory effects of PFOS on cell proliferation (Fig. 4c).

## Discussion

Breast cancer is one of the most common malignancies affecting women in Western countries (Gullick et al. 1998). Despite extensive research efforts to understand and eradicate breast cancer, the cellular processes that can lead to the onset of mammary carcinogenesis have yet to be elucidated in detail. Among many breast cancer risk factors, estrogens and non-estrogenic endocrine disruptors are considered to play critical roles in human breast carcinogenesis (Brody et al. 2007). Several common persistent organic pollutants are endocrine disrupters and suggested to play an important role in cancer etiology. These persistent and lipophilic compounds have been associated with effects relevant for development of breast cancer such as estrogenic, tumor promoting, and immunosuppressive activities (Bonefeld-Jorgensen et al. 2011).

There is growing evidence that PFOS may disrupt the endocrine system (Johansson et al. 2008; Wang et al. 2011). It has been shown to exhibit weak antagonistic ER transactivation, induce estradiol (E2) levels, and reduce testosterone levels in a cell line study (Kang et al. 2016). Moreover, a human study showed a significant inverse association between PFOS and estradiol in perimenopausal and menopausal women (Knox et al. 2011). However, the molecular mechanisms downstream these effects are still unclear.

In our present work, we have demonstrated that PFOS can induce cell proliferation in the MCF-10A breast cell line, through cell-cycle progression by altering the levels of proteins involved in cell-cycle regulation. PFOS induce cell proliferation, by down-regulation of p53, p21, and p27 and up-regulation of CDK4 levels, subsequently resulting in MCF-10A transformation. Importantly, PFOS also stimulated cell migration and invasion, illustrating its capability of inducing neoplastic transformation of human normal breast epithelial cells.

Unrestricted proliferation is a hallmark of cancer cells and is frequently associated with impaired cell-cycle regulation, and dysregulation of components involved in cell-cycle control occurs frequently in breast cancer (Cancer Genome Atlas 2012). A crucial abnormality that occurs during tumor development is the loss of control at the G1-to-S transition, which is a period during which cells decide to enter in the cell cycle on mitogenic stimuli. The progression through the G1–S phase requires phosphorylation of the retinoblastoma protein by CDK4 or CDK6 (Lundberg and Weinberg 1998). These enzymes, along with their corresponding cyclins D allow the cell to enter or not in the S phase (Berthet and Kaldis 2007). The observed PFOS induction of CDK4 levels could explain the increase in MCF-10A cell proliferation found in our studies. In line with this, deregulation of CDKs are common hallmarks in cell-cycle alterations found in tumors (Dickson 2014), and CDK4/6 has a pivotal role in the G1-to-S-phase cell-cycle transition in cancer (O’Leary et al. 2016). Moreover, CDK4 is found to be highly expressed in aggressive tumors and its expression correlate with poor overall and relapse-free survival outcomes as well as poor prognostic features of breast cancer patients, suggesting a central role for this protein in cancer development and progression (Massague 2004).

Concomitant with an increase in CDK4 levels, we found a decrease in p27, p21 and p53 levels. The protein inhibitors p27 and p21 negatively regulate the G1-to-S phase progression by binding to and inhibiting Cyclin E-CDK2 or Cyclin D-CDK4 (Sherr and Roberts 1999). Decreased expression of these cell-cycle inhibitors is associated with the promotion of tumor formation and a poor prognosis in many types of cancers (Burton et al. 2000). Low levels or loss of p27 and p21 expression is a significant predictor of reduced survival, tumor progression, and prognosis (Abbas and Dutta 2009; Catzavelos et al. 1997). Moreover, the p27 levels usually decrease during tumor development and progression (Vidal and Koff 2000), and there is considerable evidence that p27 inactivation is fundamental for development of malignancies (Loda et al. 1997). p21 expression is tightly controlled by the well-known tumor suppressor p53, involved in p53-dependent cell-cycle arrest, DNA repair, and apoptosis in response to various cellular stressors (Cmielova and Rezacova 2011). However, p53 is the most frequently mutated gene in human cancers and 15–60% of breast cancers contain a p53 mutation. In addition, loss of p53 by degradation has been reported in several common human cancers and might be associated with poor prognosis and death in breast cancer patients (Gasco et al. 2002).

Invasion and metastasis of aggressive breast cancer cells are the final and fatal step during cancer progression (McAllister et al. 2011). To form metastasis, cancer cells must escape the primary tumor site, migrate into surrounding tissues, and colonize a distant organ. The first barrier faced by invasive cancer cells is the basement membrane, a dense and rigid matrix. Using a transwell and matrigel assay, we showed that PFOS enhanced the migration and invasion capacity of MCF-10A cells. This effect could be related with the decreased levels of p27, p21, and p53, since it is well known that decreased concentrations of these inhibitors are implicated in human tumorigenesis or oncogenic progression in many human malignancies (Alkarain and Slingerland 2004).

**Fig. 5:**
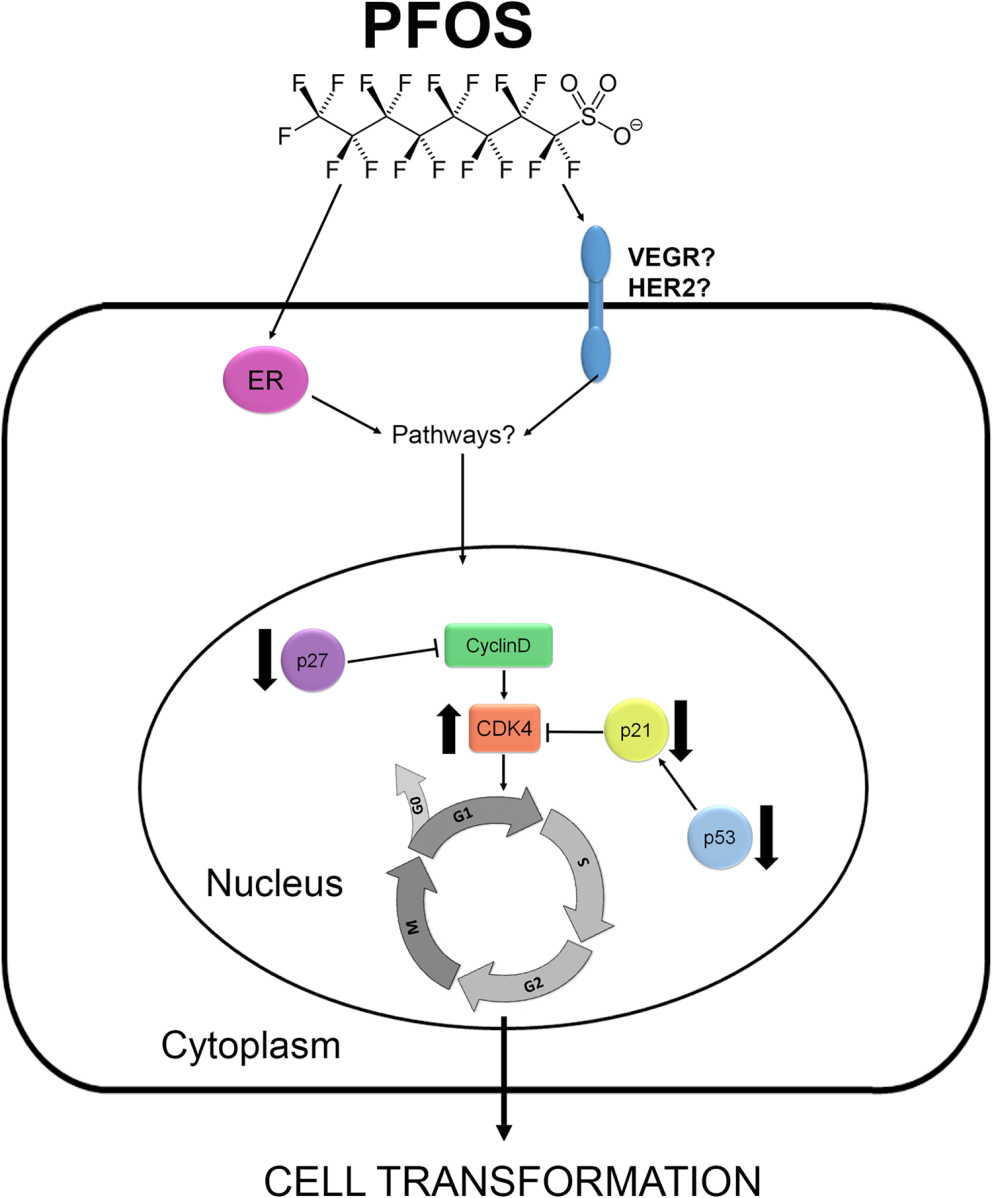
Schematic model of how PFOS may promote cell-cycle progression, and stimulate cell proliferation and transformation in MCF-10A cells. PFOS acts through ER and possibly VEGR/HER2, up-regulating CDK4 and down-regulating p27, p21, and p53, to drive cells into the cell cycle from G_1_ to S, promoting cell-cycle progression

Estrogen—a primary sex hormone responsible for mammary gland development—exerts many of its effects through the two nuclear receptors ERα and ERβ (Lee et al. 2012). In the mammary gland, ERα promotes proliferation, whereas ERβ inhibits ERα-mediated proliferation (Chang et al. 2006). Most of the human breast cancers are initially estrogen-dependent wherein ERα expression contributes significantly to the etiology of the disease (Black et al. 1983). Estrogen-activated ERα regulates the expression of several key cell-cycle regulatory genes, such as c-myc, c-fos, and cyclin D1 (Prall et al. 1997; van der Burg et al. 1989). On the other hand, studies have reported that ERβ is frequently lost during carcinogenesis, suggesting a role for ERβ as a tumor suppressor (Park et al. 2003; Skliris et al. 2003). In line with this, estrogen and estrogenic compounds can cause malignant transformation of normal breast epithelial cells through activation–expression of these two ER (Wang et al. 2013). We show that PFOS had no effect on ERα and ERβ protein expression in MCF-10A cells. However, the ER blocker ICI 182, 780 partially prevented the cell proliferation caused by the compound, suggesting that the stimulatory effects on MCF-10A proliferation—at least in part—is by activation of ER receptors. Nonetheless, other mechanisms are involved in its actions. Several molecular mechanisms have been identified which may be implicated in the loss of ER response during tumor progression. Increased expression of growth factor receptor pathways, especially EGFR/HER2, and concomitant loss of the p53, has been associated with endocrine therapy resistance (Osborne and Schiff 2011). In addition, reduced expression or activity of p21 and p27 is also associated with ER-negative breast cancer (Chu et al. 2008; Perez-Tenorio et al. 2006). Other studies have shown that pathways such as receptors for insulin/IGF1, FGF, and VEGF, as well as cellular Src, AKT, and stress-related kinases have been implicated in the transformation of ER-positive to ER-negative breast cancers (Chakraborty et al. 2010; Kern et al. 1994; Morgan et al. 2009). An overview of the proposed mechanism of PFOS toxicity is depicted in Fig. 5. However, the exact mechanisms by which PFOS are affecting the MCF-10A proliferation need to be elucidated.

In summary, PFOS significantly stimulates MCF-10A proliferation in a dose-dependent manner, which alters the levels of important proteins involved in cell-cycle regulation, subsequently resulting in cell transformation. Interestingly, MCF-10A cells also acquire the capability of migration and invasion after PFOS treatment. These effects are partially caused by activation of ER. The results suggest that PFOS is capable of transforming the human normal breast epithelial cell line MCF-10A to a malignant profile. This is the first study demonstrating PFOS-induced transformation of MCF-10A, which might be mediated by its up-regulation of CDK4 and down-regulation of p53, p21, and p27. Consequently, PFOS might play a role in breast neoplasia and breast cancer development. These results implicate PFOS exposure as a potential risk factor for breast cancer. Further experimental and epidemiological studies are warranted to determine detailed mechanisms and elucidate the role of PFOS exposure in breast cancer etiology.
